# Factors affecting the rapid changes of protein under short-term heat stress

**DOI:** 10.1186/s12864-021-07560-y

**Published:** 2021-04-13

**Authors:** Bingjin Wu, Jianwen Qiao, Xiaoming Wang, Manshuang Liu, Shengbao Xu, Daojie Sun

**Affiliations:** 1 State Key Laboratory of Crop Stress Biology for Arid Areas, College of Agronomy, Northwest A&F University, Yangling, 712100 Shaanxi China; 2 State Key Laboratory of Crop Stress Biology for Arid Areas, College of Plant Protection, Northwest A&F University, Yangling, 712100 Shaanxi China

**Keywords:** Heat stress, Posttranscriptional regulation, Codon usage, AAG frequency, Wheat

## Abstract

**Background:**

Protein content determines the state of cells. The variation in protein abundance is crucial when organisms are in the early stages of heat stress, but the reasons affecting their changes are largely unknown.

**Results:**

We quantified 47,535 mRNAs and 3742 proteins in the filling grains of wheat in two different thermal environments. The impact of mRNA abundance and sequence features involved in protein translation and degradation on protein expression was evaluated by regression analysis. Transcription, codon usage and amino acid frequency were the main drivers of changes in protein expression under heat stress, and their combined contribution explains 58.2 and 66.4% of the protein variation at 30 and 40 °C (20 °C as control), respectively. Transcription contributes more to alterations in protein content at 40 °C (31%) than at 30 °C (6%). Furthermore, the usage of codon AAG may be closely related to the rapid alteration of proteins under heat stress. The contributions of AAG were 24 and 13% at 30 and 40 °C, respectively.

**Conclusion:**

In this study, we analyzed the factors affecting the changes in protein expression in the early stage of heat stress and evaluated their influence.

**Supplementary Information:**

The online version contains supplementary material available at 10.1186/s12864-021-07560-y.

## Background

The fluctuation of protein abundance determine the state of cells, and the elements that affect protein expression have been extensively studied in recent years [1–4]. The rapid development of high-throughput technology provides technical support for research. Transcriptome sequencing is accurate and efficient, but due to the bottleneck of proteomics, it is incapable of quantifying a more comprehensive protein landscape. This discrepancy makes people usually use transcription to speculate about protein expression. Studies have shown that transcription is a weak proxy for protein abundance (*R*^2^, 0.2–0.4) in various species [5–10], especially in stressful environments (*R*^2^ < 0.09) [11–13]. In addition to the fact that protein synthesis fails to keep up with the pace of transcription, the reason for the weaker correlation under stress is that the synthesis of some proteins, such as transcription factor [14], does not depend on transcription and increases even before transcription changes [15–17]. Thus, it has been indicated that there are other regulatory mechanisms to help protein expression under stress conditions.

Although transcriptional regulation is important for protein expression, it is insufficient to represent protein variation. The genetic code, which is a template for protein synthesis, also contains information to regulate protein expression. The effects of amino acid [4], untranslated regions [18–21], length [21, 22], GC content [23–25], and mRNA secondary structure [26] have been confirmed in previous studies. Codon usage is regarded as one of the major factors in controlling elongation during translation, and the usage of preferred codons in the coding sequence can significantly increase the rate of protein synthesis [27–30]. In addition, microRNA-mediated post-transcriptional gene silencing [31] and protein degradation [32] also play an important role in regulation of protein abundance.

Previous studies have focused on the correlation between overall transcription and protein. However, when an organism is subjected to stress, most of the protein expression is stagnated, and only a small portion of the proteins bypass this inhibition and are expressed rapidly in abundance to reduce damage. The most typical example is molecular chaperones, whose expression pattern is still a mystery.

Wheat plants are sensitive to heat, especially in the middle and late stages of grain filling [33–36]. Organisms adapt to adversity by altering their protein abundance, and the factors that are related to fluctuations in protein abundance in the short term are still unclear. In this study, transcriptomic and proteomic analyses were performed on wheat grains under two types of short-term heat stress. The influencing factors of differentially expressed proteins were investigated in varying degrees of thermal environments. This study suggests that wheat grains have various response measures for different levels of heat stress and that posttranscriptional regulation plays a crucial role in regulating protein expression to adapt to constantly changing temperatures.

## Results

### Quantification of the wheat grain transcriptome and proteome under short-term heat stress

**Fig. 1:**
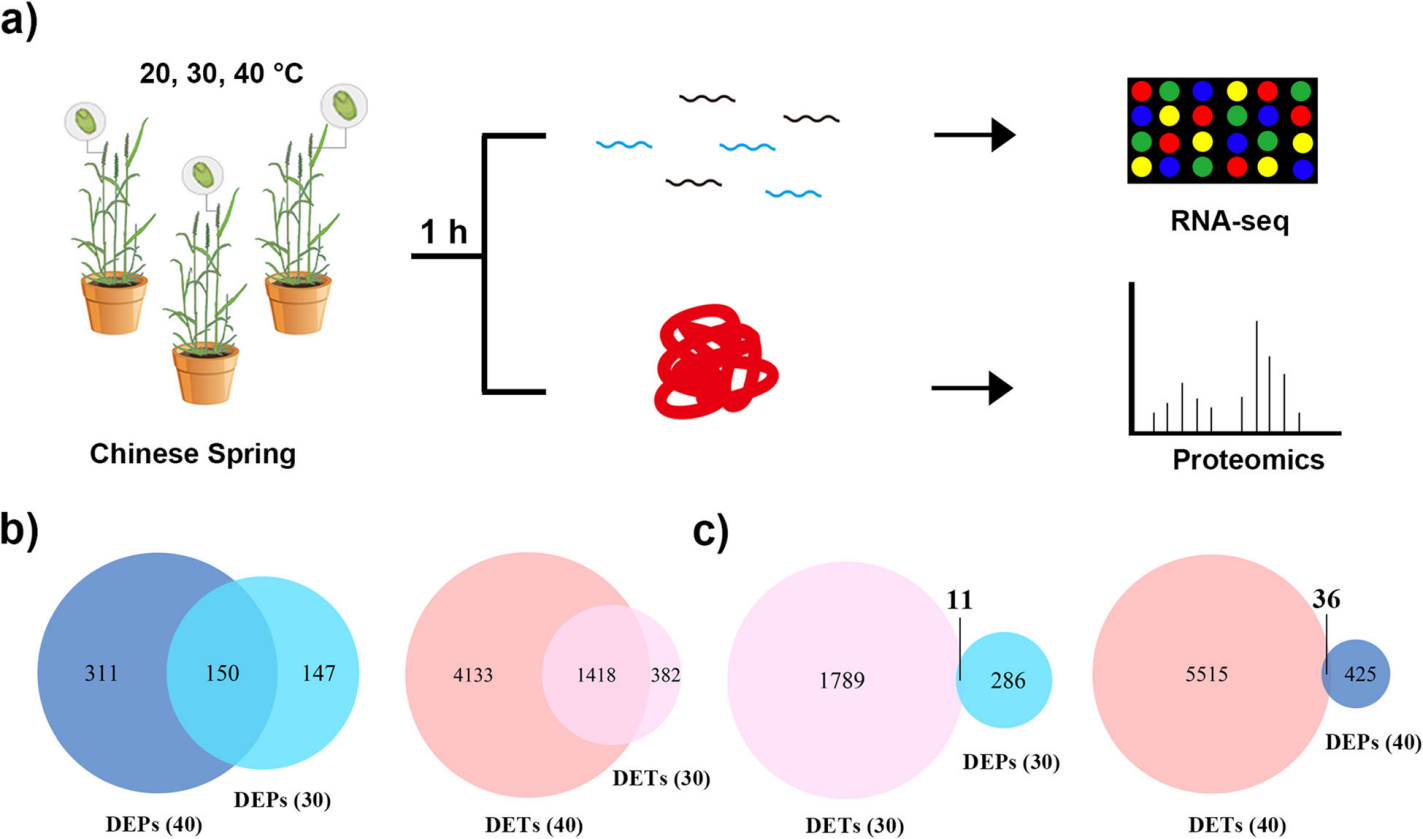
Experimental design and quantification of the transcriptome and proteome. **a** Work flow. Wheat plants were exposed to three temperatures for 1 h. Grains were quickly collected and frozen for transcriptomic and proteomic analysis. **b** Venn diagrams of differentially expressed transcripts and proteins under two heat treatments. Differentially expressed proteins (DEPs, *p* < 0.01) and transcripts (DETs, *p* < 0.05, FDR, fold change> 2 or < 1/2) were identified at the two temperatures. The number in brackets represents the respective temperature (30 °C, 40 °C), and 20 °C was used as the control. **c** Venn diagrams of differentially expressed transcripts (DETs) and proteins (DEPs) under 30 and 40 °C (20 °C as control), respectively. The number represents the overlap between DETs and DEPs

To understand how filling grain quickly adapts to temperature variations, wheat (*Triticum aestivum* cv. Chinese Spring) plants were subjected to 20, 30, or 40 °C for 1 h. The most suitable growth temperature for wheat is 12–22 °C [37], while 30 and 40 °C are different levels of heat stress [38]. We evaluated both transcriptomic and proteomic profiles of wheat grains in the three environments (Fig. 1a). A total of 47,535 transcripts were acquired (FPKM > 1 in any circumstances) via the transcriptome. Of these, 1800 and 5551 were identified as differentially expressed transcripts (DETs; fold change > 2, *p* < 0.05, FDR; Table S1) under two types of heat environments (30 and 40 °C; 20 °C served as the control). Through TMT proteomic analysis, 3742 proteins were quantified, including 297 and 461 differentially expressed proteins (DEPs; T-test, *p* < 0.01; Table S2).

More DETs and DEPs were identified under severe stress (40 °C) than under mild heat stress (30 °C), indicating that filling grains express more transcripts and proteins to respond to severe stress. In addition to 150 proteins and 1418 transcripts expressed under the two thermal treatments, wheat grains expressed specific genes and proteins in response to a certain degree of heat stress (Fig. 1b). These results revealed that grains have corresponding measures in response to various levels of heat stress.

What is striking is that there is little overlap between DETs and DEPs under short-term heat stress, whether it be mild (30 °C) or severe (40 °C) (Fig. 1c). That is, after a short-term heat stress, a small portion of the corresponding DETs and DEPs changed simultaneously, and the transcripts corresponding to most of the DEPs did not differ significantly.

### Transcription and protein levels in response to heat stress

To further study the function of transcripts and proteins that respond to heat stress in a short time, GO and KEGG enrichment analysis was performed on differentially expressed transcripts and proteins under the two types of thermal environments.

**Fig. 2:**
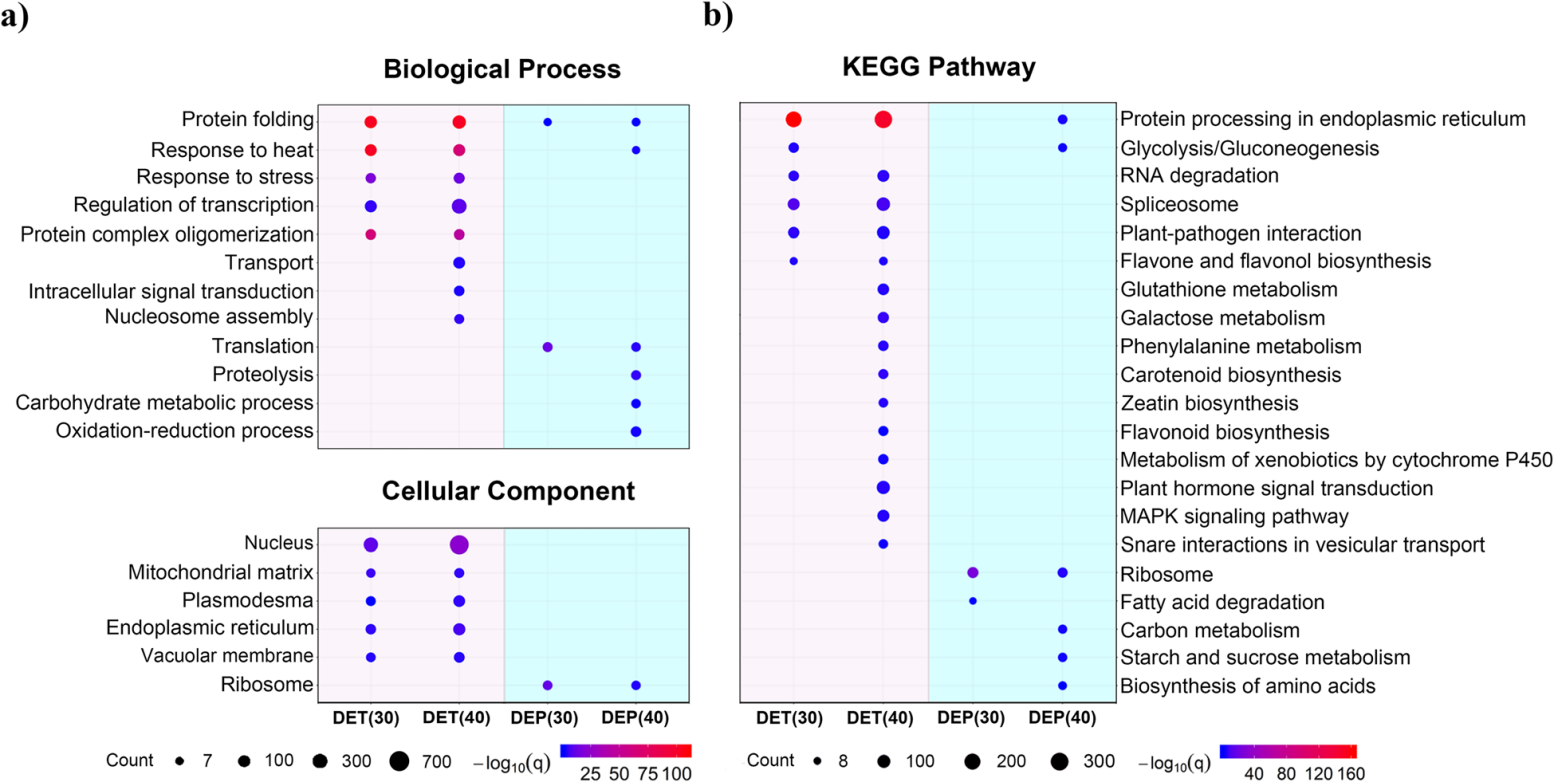
Enrichment analysis of DETs and DEPs under two types of heat stress. **a** GO enrichment. Functional enrichment analysis of DETs and DEPs associated with biological processes and cellular components under 30 and 40 °C heat treatments (*N* ≥ 7, q < 0.05). **b** KEGG enrichment. Enriched pathways of DETs and DEPs in the two types of heat environments (*N* > 7, q < 0.05)

Protein folding, one of the main ways in response to heat [39], was enriched according to GO enrichment analysis across DETs and DEPs in both treatments (Fig. 2a). Protein expression was triggered from the 1-h exposure to the thermal environment, regardless of whether it involved in mild or severe stress; the reaction speed of proteins to a disturbed environment may exceed our expectations.

More substances were enriched under severe heat stress than under mild stress at both the transcriptional and protein levels, not only increasing the number but also broadening the capability (Fig. 2). Consistent with few overlaps between DETs and DEPs (Fig. 1c), the enriched processes and pathways were also significantly different between the transcription and protein levels (Fig. 2a, b). In addition to “protein folding” and “protein processing in the endoplasmic reticulum” (Fig. 2), transcription and proteins play regulatory roles in different fields during short-term heat stress. These results imply that transcription may not be the main way to regulate the expression of proteins involved in rapid thermal responses.

As protein synthesis machine, the ribosome, was significantly enriched in the GO and KEGG analysis (Fig. 2). However, the expression of ribosomal proteins changed dramatically only at the protein level, rather than at the transcriptional level. That is to say, ribosome expression under short-term heat stress is free from transcriptional regulation or mRNA returns to undisturbed levels rapidly after translation.

### Pattern of ribosomes in response to short-term heat stress

**Fig. 3:**
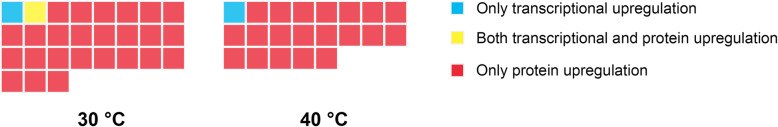
Expression patterns of differentially expressed ribosomal proteins. The differentially expressed transcripts or proteins of ribosomes were identified in 30 and 40 °C, respectively. The blue squares mean that the ribosome only changed at the transcriptional level; it did not change at the protein level. In contrast, the red squares indicate changes only at the protein level; the transcriptional level remained unchanged. The ribosomes shown in the yellow changed at both the transcriptional and protein levels

To clarify the expression patterns of ribosomal proteins under short-term heat stress, 141 ribosomal proteins identified via proteomics under two heat stresses were used for expression pattern analysis. The expression of twenty-eight and twenty-two ribosomes changed significantly at 30 and 40 °C, respectively. In addition to one or two ribosomes whose expression changed at the transcriptional level, the expression of the others increased only at the protein level (Fig. 3). Detailed information is shown in the supplementary figure (Fig. S1).

As shown in Fig. 3, ribosomal protein was expressed in large quantities, and transcription had not changed or returned to a resting state in a short time, regardless of mild (30 °C) or severe (40 °C) heat stress. This class of proteins may adopt an unknown strategy to achieve a large amount even if protein expression is suppressed.

### Factors affecting the rapid alteration of proteins under short-term heat stress

To investigate the factors affecting the variation in DEP expression under two types of short-term heat conditions, 107 factors and protein changes under two kinds of heat stress were used for regression analysis respectively. These candidate items were found to be involved in regulating protein expression in previous studies, including transcriptional alteration, codon usage, amino acid frequency, length, and a base frequency of coding sequences or untranslated regions.

We used fold change (fold change = treatment/control) as a measure to evaluate the changes in transcription and protein abundance. Through correlation analysis, in addition to the transcription, more than 20 sequence characteristics were identified as being significantly related to the alteration in protein expression (Table 1). Although transcription significantly correlated with protein variation under both treatments (0.24 at 30 °C, *p* < 0.01; 0.55 at 40 °C, *p* < 0.01), the correlation was stronger under severe heat stress. These results suggested that transcription provided greater support for altering protein expression under severe heat stress.

**Table 1:** Correlation analysis

Characteristic	P (30)	P (40)
***Transcript***		
Transcript fold change	0.24**	0.55**
***Amino Acid Frequency***		
A	−0.22**	0.09
C	−0.11	−0.24**
D	−0.30**	−0.16**
E	0.03	0.26**
K	0.48**	0.39**
P	−0.27**	−0.06
R	0.34**	0.21**
S	−0.20**	−0.22**
Y	0.10	−0.22**
***Codon Usage***		
TAC (Y)	0.06	−0.22**
GAC (D)	−0.27**	−0.1
AAG (K)	0.48**	0.40**
AAA (K)	0.02	0.05
GAG (E)	0.07	0.30**
CCG (P)	−0.27**	−0.10
AGC (S)	−0.30**	−0.21**
TGC (C)	−0.09	−0.22**
CGC (R)	0.18**	0.20**
AGG (R)	0.29**	0.11*
CGT (R)	0.39**	0.12*
***Base Frequency***		
A (CDS)	0.20**	0.15**
T (3’UTR)	0.25**	0.23**
GC (5’UTR)	0.27**	0.15**

Factors related to protein expression at two heat treatment. P (30) and P (40) represent fold changes at 30 °C and 40 °C, respectively. ** The correlation is significant at the 0.01 level (2-tailed). * The correlation is significant at the 0.05 level (2-tailed)

Interestingly, the frequency of lysine was very strongly correlated with protein variation, even more so than transcription was (Table 1). Lysine is encoded by two codons, AAG and AAA. AAG was also associated with protein changes (0.48 at 30 °C, *p* < 0.01; 0.40 at 40 °C, *p* < 0.01), while AAA was not. It is easy to determine that the correlation coefficient between AAG frequency and protein alteration was nearly the same as that of lysine. Namely, AAG usage, rather than lysine frequency, is one of the major factors regulating changes in protein abundance. In addition, several codons, amino acids, and base frequency also showed a strong relationship with protein variation in different situations. Sequences containing more factors which positively associated with protein expression and less negatively associated factors are more likely to respond to short-term heat stress.

### Contribution of factors to protein expression under heat stress

**Fig. 4:**
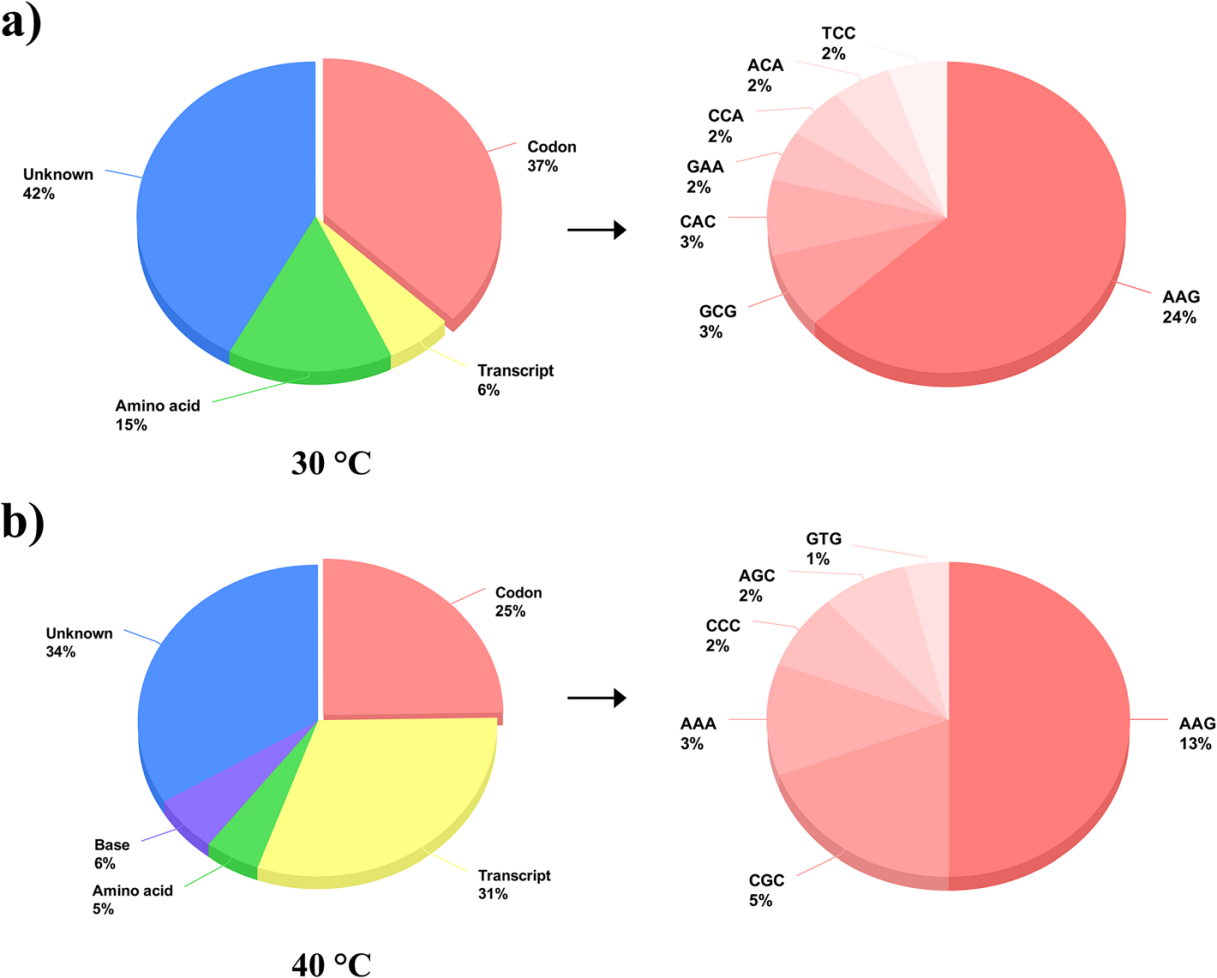
Contributions of various factors to protein expression under two types heat stress. **a** and **b** Factors and their contributions to protein expression under 30 and 40 °C. By analyzing transcriptome data, proteome data and 107 sequence characteristics, the factors affecting protein expression and their contributions were shown on the pie chart under 30 and 40 °C, respectively. The pie on the right is an expanded view of the codon usage

To explore the underlying mechanism of the adjustment of heat-responsive proteins in two types of heat stress, a regression analysis of the 107 factors was performed to evaluate their contribution. We applied multivariate adaptive regression spline (MARS) to fit the model and calculate the importance of each variable under two thermal environments, and linear regression and elastic net were used for verification. The equations fitted by the factors explained 58.2 and 66.4% of the changes in protein under mild (30 °C) and severe (40 °C) heat stress, respectively (Fig. 4, Table S3). Codon usage, transcription, and amino acid frequency had the most significant impact on protein expression.

Limited contribution (6%) of transcription to protein changes occurred under mild heat stress (30 °C); however, transcription had a dramatic effect (31%) on protein alteration when plants were under extreme heat stress (40 °C) (Fig. 4). These findings showed that transcriptional regulation may have a marginal impact under mild stress, but have a significant role in the acute thermal response.

Interestingly, codon usage largely affected protein expression (37% at 30 °C; 25% at 40 °C), whether under mild or severe heat stress, indicating that codon preference strongly supports the rapid variation in proteins under heat stress. In line with the correlation results (Table 1), the codon AAG may play an important role in the rapid alteration of proteins under heat stress. The contributions of AAG were 24 and 13% at 30 and 40 °C, respectively (Fig. 4). Although the lysine content was also significantly correlated with variation of protein expression, the frequency of lysine (K) was removed from the equation after MARS analysis. That is, the critical function for rapid protein expression is AAG instead of lysine. Similar results were also obtained by linear regression and elastic net (Table S4), supporting the credibility of the equation.

When an organism responds to environmental disturbances, only transcriptional regulation may be too slow. The mechanism of regulating protein expression is written in the sequence, which may be the fastest and most effective response mode.

### The relationship between protein expression and the AAG occurrence frequency under short-term heat stress

**Fig. 5:**
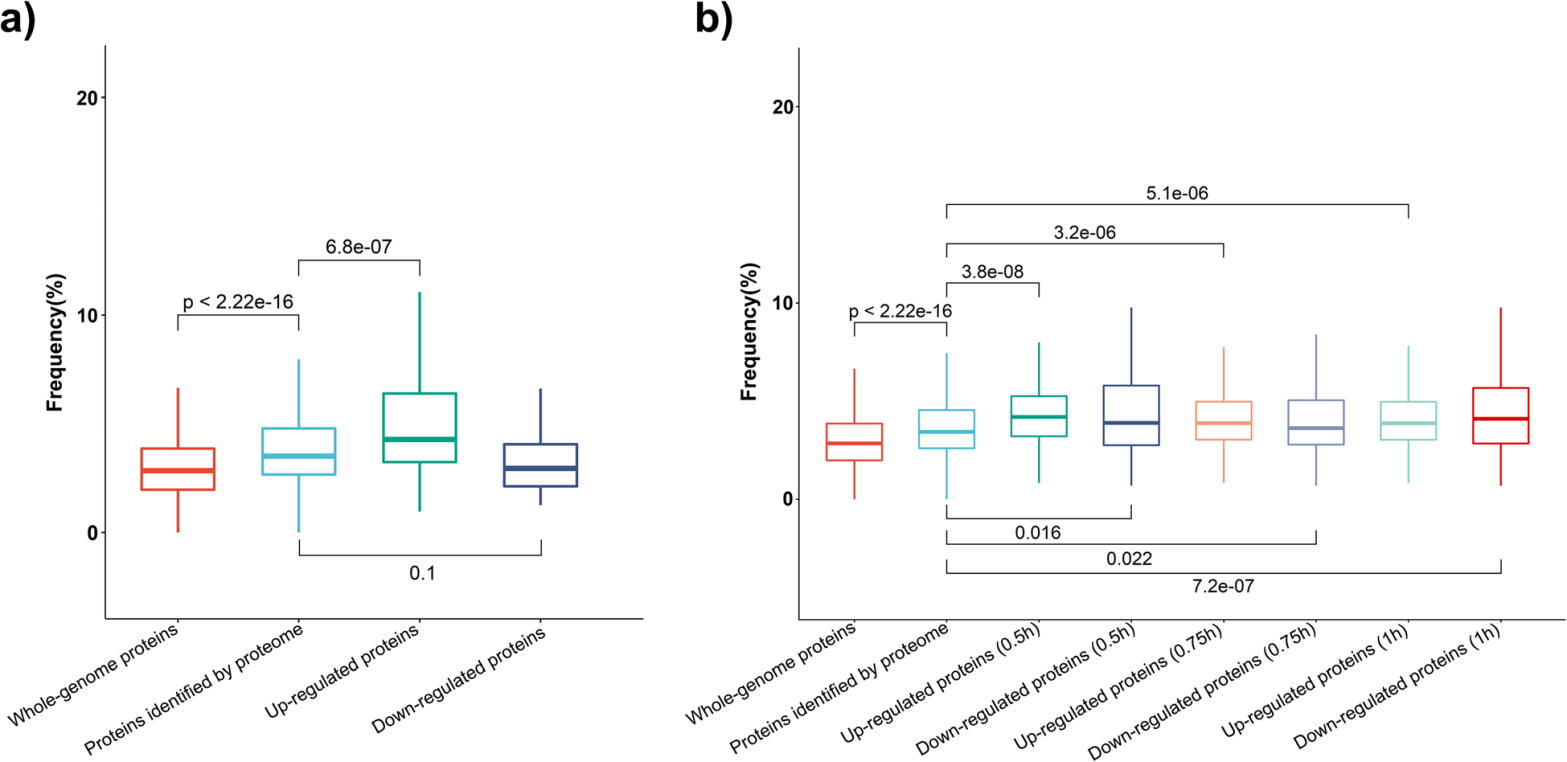
Frequency of AAG codon in different groups of proteins under short-term heat stress. **a** Distribution of AAG codon occurrence frequency for different groups of genes in yeast strain BY4742 subjected to short-term heat stress. The proteome data used in this analysis was published by the previous study [40]. Yeast was cultured at 30 °C was taken as control. Heat stress treatment was applied by transferring yeast to 37 °C for 1 h. Up and down-regulated proteins in response to heat treatment were identified with criteria “FDR < 0.05 and fold change >1.2 or < 0.83”. The Y-axis represents the frequency of codon AAG in protein-coding sequence. *P*-values were calculated by the Kruskal-Wallis method. **b** Distribution of AAG codon occurrence frequency for different groups of genes in yeast strain BY4741 subjected to short-term heat stress. The proteome data used in this analysis was published by the previous study [41]. Yeast strain BY4741 cultured at 25 °C was taken as control. For heat treatment, yeast was exposed to 37 °C for 5, 10, 15, 30, 45 and 60 min. The criteria for up-regulated and down-regulated proteins are identified by adjusted *p* < 0.01 and fold change >1.2 or < 0.83. The numbers in brackets represent the heat treatment time

To verify whether the up-regulated proteins under short-term heat stress are rich in AAG codon, two previously published proteomic datasets of yeast subjected to heat stress were analyzed [40, 41]. The AAG codon occurrence frequencies were calculated for the transcripts corresponding to the whole-genome proteins (all the proteins annotated in yeast genome), the proteins identified by proteome analysis and the differentially expressed proteins under heat stress (Fig. 5a, b).

Compared with the whole-genome proteins, higher AAG usage was observed in the coding sequence of protein identified in the proteomic analysis (Fig. 5a, b). Furthermore, the transcripts encoding up-regulated proteins under short-term heat stress have significantly higher AAG frequency than that of the proteins identified in the proteome analysis, which indicated that codon AAG might play an important role in the rapid expression of proteins responsive to heat stress (Fig. 5a, b).

## Discussion

Here, we performed transcriptome and proteome sequencing of filling grains subjected to different degrees of heat stress (30 °C, 40 °C) for 1 h. Transcription and elements related to protein expression were investigated for rapid protein variation under two types of short-term heat stress.

### Factors affecting protein expression

In recent decades, the contribution of transcription to protein has been widely discussed in different kingdoms and environmental conditions. Yeast is one of the simplest eukaryotic organisms. In yeast, transcription determined about 60% of protein expression [1, 42]. In mice and humans, 40 and 27% of protein expression were controlled by transcription [6, 43, 44], respectively. It is indicated that the more complex the organism, the more limited the role of transcriptional regulation.

Previous studies mainly focused on the relationship between total transcription and protein. However, our research has concentrated on the factors that affect the expression of changed proteins under disturbed conditions. Therefore, the contribution of transcription to protein expression depends on the degree of stress. We evaluated the contribution of transcript abundance changes to protein expression under various degrees of heat stress. The transcriptional regulation of proteins under 40 °C (31%) was more intense than that under 30 °C (6%). The posttranscriptional regulation always played a stable role (36–52%). Posttranscriptional regulation played a more important role than did transcription under mild stress, while under severe heat stress, transcription and posttranscriptional regulation worked synergistically to express the desired protein faster and stronger.

In previous studies, researchers often used the codon adaptation index (CAI) to characterize the impact of codons on translation, which explain up to 6% of protein expression [1]. We evaluated the effect of each codon on protein changes under heat stress, explaining 25–37% of protein changes. The impact of codons on protein expression may be beyond our knowledge. The usage of amino acids is rarely involved in previous studies. Our research indicated that the usage frequency of amino acids may also be related to protein expression.

### Ribosomal protein in response to heat stress

Protein synthesis is the most time- and energy-consuming process in organisms. If something goes wrong, the consequences can be catastrophic, especially under adverse conditions. Therefore, as protein synthesis machines, ribosomes need to adjust their production plan in time to quickly express the required proteins to reduce the damage caused by stress.

A total of 141 ribosomal proteins were identified via proteomics, among which 28 and 22 were up-regulated under two types of short-term thermal stress (30 °C and 40 °C, respectively). Due to the limitation of proteomics technology, ribosomal proteins with altered expression should be amplified proportionately. Approximately 14–20% of ribosomes are involved in the translation of heat-responsive proteins. This feature of ribosomes involves the preferential translation of a subset of functionally-related mRNAs and has been a popular research topic in recent years [45–48]. Heterogeneous ribosomes facilitate the synthesis of stress response proteins and help cells cope with environmental changes.

As shown in Fig. 3, a portion of the upregulated ribosomal protein expression may not be associated with transcriptional alteration. This pattern of regulation of ribosomes is often observed in cancer and stress research. In the study of rhabdomyosarcoma, it was found that the abundance of eL36 and eL42 (60S ribosomal protein L36 and L42) increases, while the corresponding mRNA decreases [49]. Several ribosomal proteins, such as CAC1787 (30S RPS2), CAC3105 (30S RPS4), CAC3147 (50S RPL1) and CAC3132 (50S RPL4), are also disconnected from transcription under butanol stress in *Clostridium acetobutylicum* [50]. The short-term adaptation of cells to new states usually requires the involvement of posttranscriptional mechanisms, because transcriptional regulation alone would be too slow. High-level translation of existing transcripts can help to synthesize needed proteins rapidly, while the targeted degradation of proteins can accelerate the removal of unnecessary proteins [16]. This adjustment by the organism can lead to quick adaptations in response to environmental disturbances.

### Codon-based regulation of protein expression

From the regression results (Fig. 4), changes in codon use were more closely related to protein expression than transcription. Among codons, AAG (Lys) is the most remarkable, contributing 24 and 13% in the two thermal environments.

Involvement of the AAG codon has been shown in many studies. By the use of the model to predict the reaction of proteins after doubling the codon usage, AAG was shown to have the most significant effect on increased protein expression in human tissue [51]. All codons encoding lysine are replaced with AAA or AAG, and the protein expression before and after replacement is observed and compared under heat stress. It can be used to test whether AAG can be quickly translated under heat stress. The results of this experiment will be interesting.

The selection of AAG by heat-responsive proteins is also related to tRNA modification. m1A-modified tRNA has a higher affinity for the elongation factor EF1A (elongation factor 1-alpha), which delivers tRNA to the ribosome [52]. ALKBH1 (histone H2A dioxygenase) is an RNA demethylase that mediates the removal of the methyl group from the N1-methyladenosine (m1A) in tRNA. tRNA^Lys^_CUU_, which complementarily pairs with AAG in the coding sequence, is one of the most important binders of ALKBH1 [53]. Therefore, tRNA^Lys^_CUU_ is widely modified by m1A to promote translation efficiency. Studies have shown that heat shock significantly increases m1A levels [54], so that when an organism is subjected to heat stress, a AAG-rich sequences are translated efficiently.

On the other hand, tRNA^Lys^_UUU_ needs to be modified with mcm^5^s^2^U^34^, an important form of post-transcriptional modification of tRNA, to maintain efficient translation of AAA codons. Studies have indicated that this modification occurs at a low level under high temperature, leading to stagnant translation of AAA-rich sequences, while AAG does not require this modification [55–57]. In addition, the continuous codon AAA easily causes ribosome sliding and premature termination [58]. Therefore, the expression of AAA-rich sequences will slow down or stagnate at high temperatures and may not participate in the thermal response. Genes related to ALKBH1 and mcm^5^s^2^U^34^ are overexpressed and the expression of sequences is observed which rich in AAG and AAA. Thus, it can be judged whether tRNA modification is involved in participating in determining which codon translates fastest under heat stress.

Above all, the codon AAG may be chosen as a means to quickly and easily identify whether mRNA is highly expressed in a thermal environment.

### AAG-rich genes

We enriched the genes with a high frequency of AAG (frequency > 0.08) in the whole wheat genome (Fig. S2); nucleosomes, ribosomes, and molecular chaperones were significantly enriched.

Half of the ribosomes and the vast majority of nucleosomes have an abundance of AAG presence, while only 15% of molecular chaperones do. Ribosomes rich in AAG have been confirmed [59], and they usually have a shorter sequence and are in an active transcriptional state. This is conducive to the use of existing mRNA for efficient expression after environmental disturbance.

In addition to posttranscriptional regulation of molecular chaperone expression, transcriptional regulation also plays an important role. Unlike ribosomes, chaperones do not have an enormous presence and needs a large amount in a short time to help misfolded proteins. This explains the mystery that has been unresolved for a long time how heat shock proteins quickly respond to heat stress.

In conclusion, through a systematic analysis of the factors of changes in protein expression under heat stress, the related factors of protein expression and their influence have been described under short-term heat stress. High expression of housekeeping and heat-responsive genes may have solved evolutionarily the problem of rapid expression under heat stress by increasing the ratio of AAG.

## Conclusions

By analyzing the transcriptome and proteome data under two kinds of heat stress, the factors were revealed which affect the rapid expression of proteins. Codon usage may play an important role in the rapid translation of proteins, especially for AAG. Moreover, the ability of transcriptional regulation changed according to the degree of heat stress. Transcription and post-transcriptional regulation worked synergistically to express the desired protein faster under heat stress. Our study revealed the main factors affecting the changes of protein expression in the short-term heat stress and explained the potential mechanism that heat-responsive protein expressed rapidly under heat stress.

## Methods

### Plant materials and growth conditions

Chinese Spring (*Triticum aestivum* L.) is thought to be a Sichuan variety. The wide application of this variety and its derived genetic stocks has greatly advanced wheat genetics, including the recent achievement of genome sequencing of wheat.

Wheat seeds (Chinese Spring) was grown in a greenhouse, and daily care was taken to avoid stress. The main stem of the plant was labeled when the first flower appeared on the spike. Twelve days after flowering, they were transferred to growth chambers with a temperature of 20 °C, and a 14/10 h day/night photoperiod for 3 days of adaptation; all the plants were in good condition. All the plants were divided into three groups (approximately 50 plants); the plants in two groups were quickly transferred to incubators that were preheated to 30 °C and 40 °C, and the plants in the other group remained at 20 °C as the control. The grains on the main stem were collected at the same time after exposure to three temperature environments for 1 h (in light), immediately frozen in liquid N_2,_ and stored at − 80 °C for transcriptomic and proteomic analysis.

### RNA sequencing

Grains from three independent plants in each treatment were taken for mRNA sequencing. The RNAs of 9 samples were subjected to 150 bp paired-end sequencing using the Illumina HiSeq X Ten platform. The sequencing depth is 10X. Trimmomatic (version 0.36) [60] was used to remove the adapters and filter low-quality reads and bases from the next-generation sequencing (NGS) data. The RNA-seq reads were aligned to the wheat reference genome, IWGSC v1.0 [https://urgi.versailles.inra.fr/download/iwgsc/IWGSC_RefSeq_Assemblies/v1.0/]. FPKM (fragments per kilobase per million) were calculated using RSEM version 1.3.0 [61] and edgeR version 3.24.3 [62] were used to identify the differentially expressed transcripts. Scripts for processing NGS data have been uploaded to GitHub (https://github.com/shangguanhehe/NGS.git).

### Isobaric tandem mass tag (TMT)-labeled quantitative proteomics

A total of 12 samples (four biological replicates each) under the three types of treatment were quantified via proteomics. After wheat grain proteins were extracted, they were digested in a solution with trypsin and labeled with a TMT isobaric mass tagging kit (Thermo Fisher Scientific). The method of protein extraction was based on Wang et al. [63]. The mixture was then physically separated by high-performance liquid chromatography (HPLC) and further analyzed for peptides using mass spectrometry (MS).

The resulting MS/MS spectra were processed using the MaxQuant search engine (version 1.5.2.8). Tandem mass spectra were searched against the wheat protein database (https://urgi.versailles.inra.fr/download/iwgsc/IWGSC_RefSeq_Annotations/v1.1/iwgsc_refseqv1.1_genes_2017July06.zip) concatenated with the reverse decoy database. Trypsin/P was specified as a cleavage enzyme allowing up to 2 missings. The first search and main search range were set to 5 ppm, and 0.02 Da of fragment ions. Carbamidomethylation on Cys was specified as a fixed modification, and oxidation on Met, and oxidation on Met and acetylation on the protein N-terminus were specified as variable modifications. False discovery rate (FDR) thresholds for protein, peptide, and modification sites were specified at 1%.

Three or more identified proteins among the four biological replicates were considered as reliable quantitative data. The missing values were filled with averages of the other three and normalized.

### Bioinformatic analysis

Hypergeometric distributions (phyper, R) were used for GO and KEGG enrichment analysis to test the significance of items; furthermore, the *p*-value was adjusted by the false discovery rate (FDR) to reduce the probability of false positives. The entries were chosen with a sufficiently large count (*N* ≥ 7) and a suitable q-value (FDR < 0.05). Annotations of wheat genes were obtained from the URGI website (https://urgi.versailles.inra.fr/download/iwgsc/IWGSC_RefSeq_Annotations/v1.0/).

### Factors affecting protein expression

Transcription and sequence characteristics were used to analyze the effects on protein expression under thermal conditions. Protein sequences, coding sequences (CDS), and annotation files were obtained from the website (https://urgi.versailles.inra.fr/download/iwgsc/IWGSC_RefSeq_Annotations/v1.0/), and untranslated region (UTR) were extracted from the genome annotation gff3 file. Sequence features such as the length of the sequences, base, codon, and amino acid usage frequency were obtained via python scripts (https://github.com/shangguanhehe/Systems-biology.git). Transcript and protein fold changes were obtained from transcriptome and proteome identification and the subsequent data analysis.

### Multivariate adaptive regression splines (MARS)

Multivariate adaptive regression splines (MARS) was used to assess the individual and combined contribution of the selected features to protein fold changes. MARS is a statistical technique for modeling data and it is an extension of linear regression that captures nonlinearities and interactions between variables [64]. MARS analysis was implemented via the ‘earth’ package (version 5.1.2) on the R platform. To make the results more reliable, linear regression (IBM SPSS, version 22) and elastic net (R, glmnet 2.0–18) were used for verification.

## Supplementary Information

**Additional file 1: Figure S1.** Changes in the transcription and protein levels of 141 ribosomes identified in two thermal environments. T30, T40, P30 and P40 represent transcriptional changes at 30 °C, transcriptional changes at 40 °C, protein changes at 30 °C and protein changes at 40 °C, respectively.

**Additional file 2: Figure S2.** Function of codon-rich AAG genes. **a)** Among the whole-genome data of wheat, genes with an AAG frequency >0.08 in the coding sequence were subjected to enrichment analysis. **b)** GO enrichment of AAG-rich genes (*N* > 60, q < 1e-20).

**Additional file 3: Table S1.** Identification of DETs.

**Additional file 4: Table S2.** Identification of DEPs.

**Additional file 5: Table S3.** MARS regression results.

**Additional file 6: Table S4.** Linear and elastic net regression.

## Data Availability

The RNA-seq datasets used in this study are deposited under the NCBI accession number GSE157909. The MS data were deposited into the ProteomeXchange Consortium via the PRIDE database [65] partner repository under the dataset identifiers PXD021460.

## Abbreviations

FPKM: Fragments per kilobase per million
DET: Differentially expressed transcript
DEP: Differentially expressed protein
MARS: Multivariate adaptive regression spline
TMT: Tandem mass tag
MS: Mass spectrometry
FDR: False discovery rate
