# Supplementary figures and images for: Factors affecting the rapid changes of protein under short-term heat stress
Source: BMC Genomics. 2021 Apr 13;22:263. doi: 10.1186/s12864-021-07560-y (PMC8042900; doi:10.1186/s12864-021-07560-y)

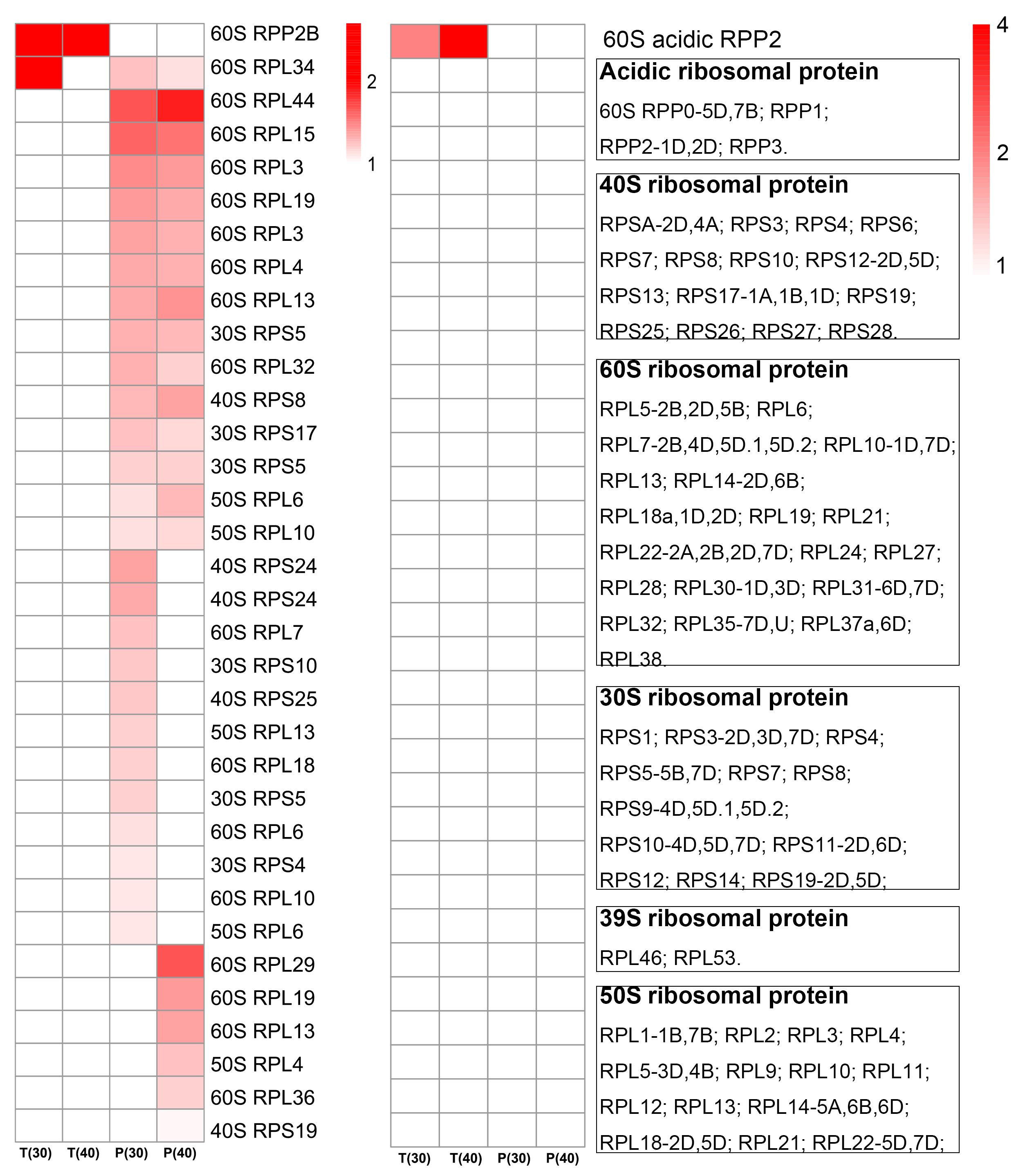

Supplement: Supplementary file 1 — Additional file 1: Figure S1. Changes in the transcription and protein levels of 141 ribosomes identified in two thermal environments. T30, T40, P30 and P40 represent transcriptional changes at 30 °C, transcriptional changes at 40 °C, protein changes at 30 °C and protein changes at 40 °C, respectively. [file 12864_2021_7560_MOESM1_ESM.tif]

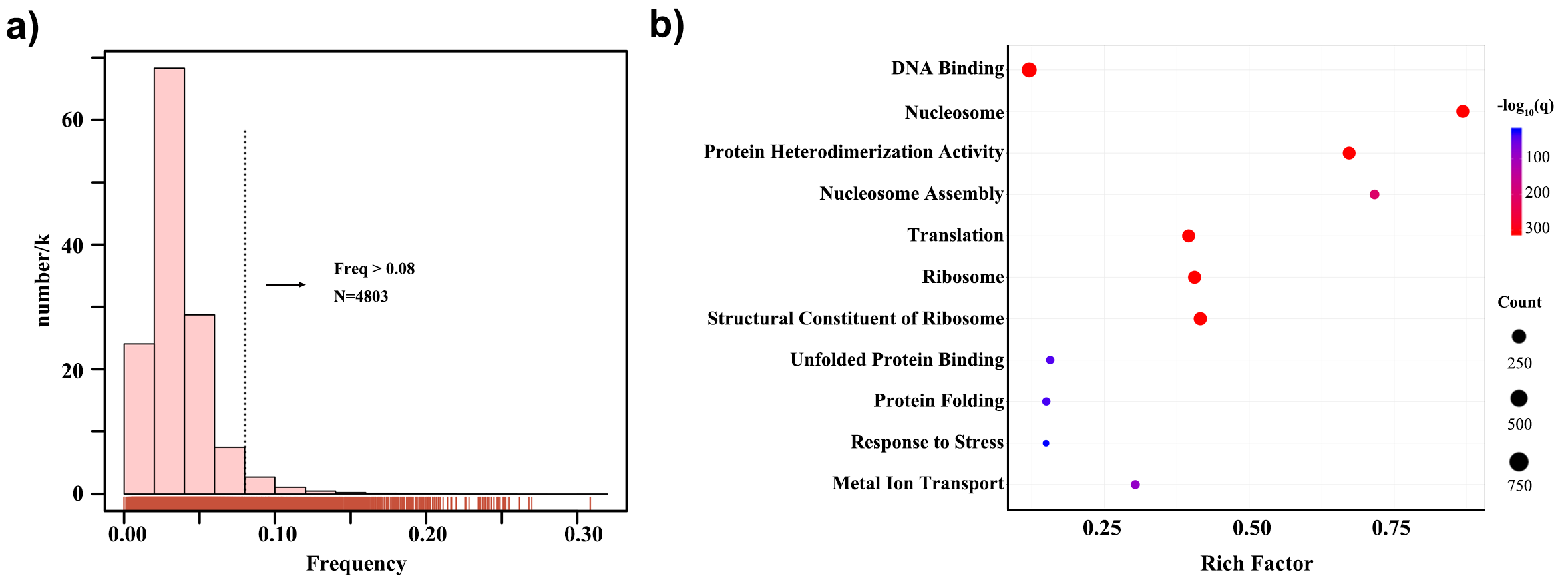

Supplement: Supplementary file 2 — Additional file 2: Figure S2. Function of codon-rich AAG genes. a) Among the whole-genome data of wheat, genes with an AAG frequency >0.08 in the coding sequence were subjected to enrichment analysis. b) GO enrichment of AAG-rich genes (N > 60, q < 1e-20). [file 12864_2021_7560_MOESM2_ESM.tif]
